# Kinesin-14 motor protein KIFC1 participates in DNA synthesis and chromatin maintenance

**DOI:** 10.1038/s41419-019-1619-9

**Published:** 2019-05-24

**Authors:** Ya-Lan Wei, Wan-Xi Yang

**Affiliations:** The sperm laboratory, College of Life Sciences, Zhejiang University, Hangzhou, Zhejiang China

**Keywords:** Chromosome segregation, Protein translocation

## Abstract

The nuclear localization signal (NLS) in kinesin-14 KIFC1 is associated with nuclear importins and Ran gradient, but detailed mechanism remains unknown. In this study, we found that KIFC1 proteins have specific transport characteristics during cell cycle. In the absence of KIFC1, cell cycle kinetics decrease significantly with a prolonged S phase. After KIFC1 overexpression, the duration of S phase becomes shorten. KIFC1 may transport the recombinant/replicate-related proteins into the nucleus, meanwhile avoiding excessive KIFC1 in the cytoplasm, which results in aberrant microtubule bundling. Interestingly, the deletion of *kifc1* in human cells results in a higher ratio of aberrant nuclear membrane, and the degradation of lamin B and lamin A/C. We also found that *kifc1* deletion leads to defects in metaphase mitotic spindle assembly, and then results in chromosome structural abnormality. The *kifc1*^*-/-*^ cells finally form micronuclei in daughter cells, and results in aneuploidy and chromosome loss in cell cycle. In this study, we demonstrate that kinesin-14 KIFC1 proteins involve in regulating DNA synthesis in S phase, and chromatin maintenance in mitosis, and maintain cell growth in a nuclear transport-independent way.

## Introduction

Kinesin-14 KIFC1 transports various cargos along the microtubule to the minus ends^1^. The conserved C-terminal motor domain of KIFC1 contains the microtubule-binding site and a catalytic region for the hydrolysis of ATP^2,3^. In addition, with the regulation of stalk domain, the rotation and steps of transportation were determined^2–5^. KIFC1 tail domain can specifically recognize and bind to the membranous organelles^6^, vesicles and γ-tubulin^7,8^, and it participates in microtubule assembly and crosslinking^9^. Besides, there is a conserved nuclear localization signal (NLS) in the tail domain of *kifc1*, similar to chromokinesins (kinesin-4 and kinesin-10), which allows kinesin motors transferring into the nucleus in interphase, but without the basic leucine zipper DNA-binding motif^10,11^. During cell mitosis, KIFC1 mainly cluster the spindles involving in chromosome alignment and segregation. While chromokinesins interact with condensins and link chromosomes to the spindles for chromosome congression and movement^12,13^.

The nuclear pore complex (NPC) is a unique transporter for nucleo-cytoplasm transportation^14^. Large protein (>45 kDa) containing the NLS can be translocated into the nucleus through the Ran GDP/GTP gradient between nucleus and cytoplasm^15–17^. Classical NLS directly combines with importin β (also known as Kap β) and targets to the NPC^18^. During mitosis, the phosphorylation of NLS also regulates spindle assembly through the importin/Ran system^19^. The NLS is generally composed of a continuous or separated in two motifs by 10 redundant amino acids^20,21^. Human KIFC1 protein belongs to the latter, with a conserved sequence KRX10-12K(K/R)(K/R)^22^. After site-specific NLS mutation, KIFC1 proteins completely accumulate at the cytoplasm and lead to excessive microtubule assembly^23^. This is because the importin α/β complex has a strong affinity for NLS, which can competitively perturb the functions of microtubule-binding site, thus results in KIFC1 lose the ability to crosslink microtubules^24^. One of the hypotheses is that the main purpose of nuclear transportation is to maintain proper assembly of microtubules in interphase and avoid abnormal microtubule crosslink, which leading to disordered cytoskeleton in interphase. Moreover, besides of the degraded DNA diffusing in the cytoplasm, there are many other naked DNA molecules that can be transported into the nucleus and integrated into the genome. During interphase, KIFC1 proteins can specifically bind to and transport these DNA molecules, whereas chromokinesins cannot^25^. Thus, another hypothesis holds that KIFC1 mainly transports the bare exogenous DNA into the nucleus. Similarly, KIFC1 mostly dispersed in the cytoplasm and transported into the nucleus before mitosis when centrosome separation occurred^26^. This suggests that KIFC1 has independent functions in different phases in cell cycle. Therefore, it’s important to clarify the distinct translocation of KIFC1 proteins and its specific functions in the nucleus and in cell cycle.

KIFC1 also has independent functions in germ cells^27–30^, which generally overexpressed in a variety of cancer cells, and is even positively correlated with the stage and malignancy of tumor. In cancer cells, KIFC1 proteins are disrupted by a higher level of Ran and form multipolar spindles with numerous clustered centrosomes, thus it’s considered as one of the targeted proteins of cancer cells for clinical usage^31–33^. Multiple small molecules were screened for inhibiting the KIFC1 function specifically^34,35^. However, it is unknown that whether the ablation of KIFC1 in normal cells is unnecessary or can be replaced by other proteins in cells.

In this study, we used two *kifc1*^*−/−*^ cell lines which knockout different *kifc1* loci to determine the functions of KIFC1 during cell cycle. Here, we revealed that the ablation of KIFC1 proteins in human cells cause cell growth inhibition, reduced cell cycle kinetics, deformed cell membrane, chaotic chromatin density and aneuploidy.

## Results

### KIFC1 proteins mainly translocate into nucleus during S phase

**Fig. 1:**
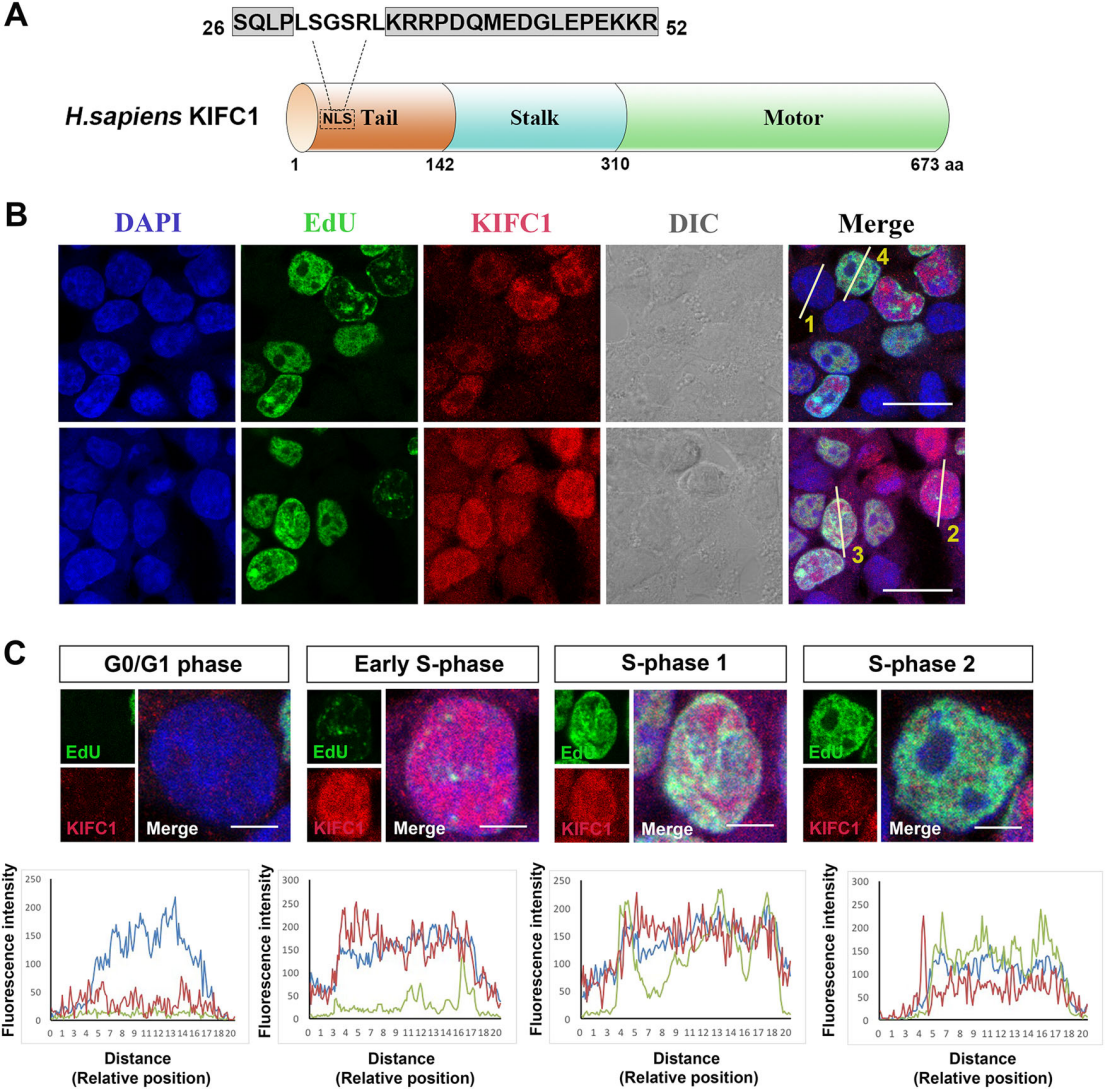
KIFC1 proteins translocate into nucleus during DNA replication. **a** Graphical model of human KIFC1 with the major three domains: motor domain, stalk domain and tail domain. Specifically, NLS is a conserved sequence (grey label) of tail domain for nuclear translocation. **b-c** The spatio-temporal positioning of KIFC1 (red) during cell cycle. EdU (green) incorporated into DNA to distinguish the replication period from G1 phase to the late S-phase (indicated separately by 1–4) with the corresponding fluorescence intensity and relative cell position measurement. The blue lines (DAPI) represent to the nucleus, and the red lines indicate the relative localization of KIFC1. DIC (Differential Interference Contrast). Scale bars = 5 µm

NLS in the tail domain of KIFC1 is a crucial motif for nuclear translocation (Fig. 1a). To clarify the function of KIFC1 in nucleus, we used 5-ethynyl-2′-deoxyuridine (EdU) incorporated into DNA to distinguish the specific period of interphase when KIFC1 is involved in nuclear localization in the cell cycle. Here, we recorded multiple cells and measured the fluorescence intensity of KIFC1 and relative positions using a laser scanning confocal microscopy (Fig. 1b). At interphase cells, KIFC1 proteins were dispersed throughout the cells without specific localization. Surprisingly, many KIFC1 proteins entered the nucleus at the very beginning of DNA synthesis, while they gradually translocated out of the nucleus at the end of the S phase (Fig. 1c). Taken together, KIFC1 may function in DNA synthesis with a nuclear translocation characteristic mainly reflected during S phase, especially when the DNA synthesis was just occurred.

### KIFC1 is essential for cell growth and proliferation

**Fig. 2:**
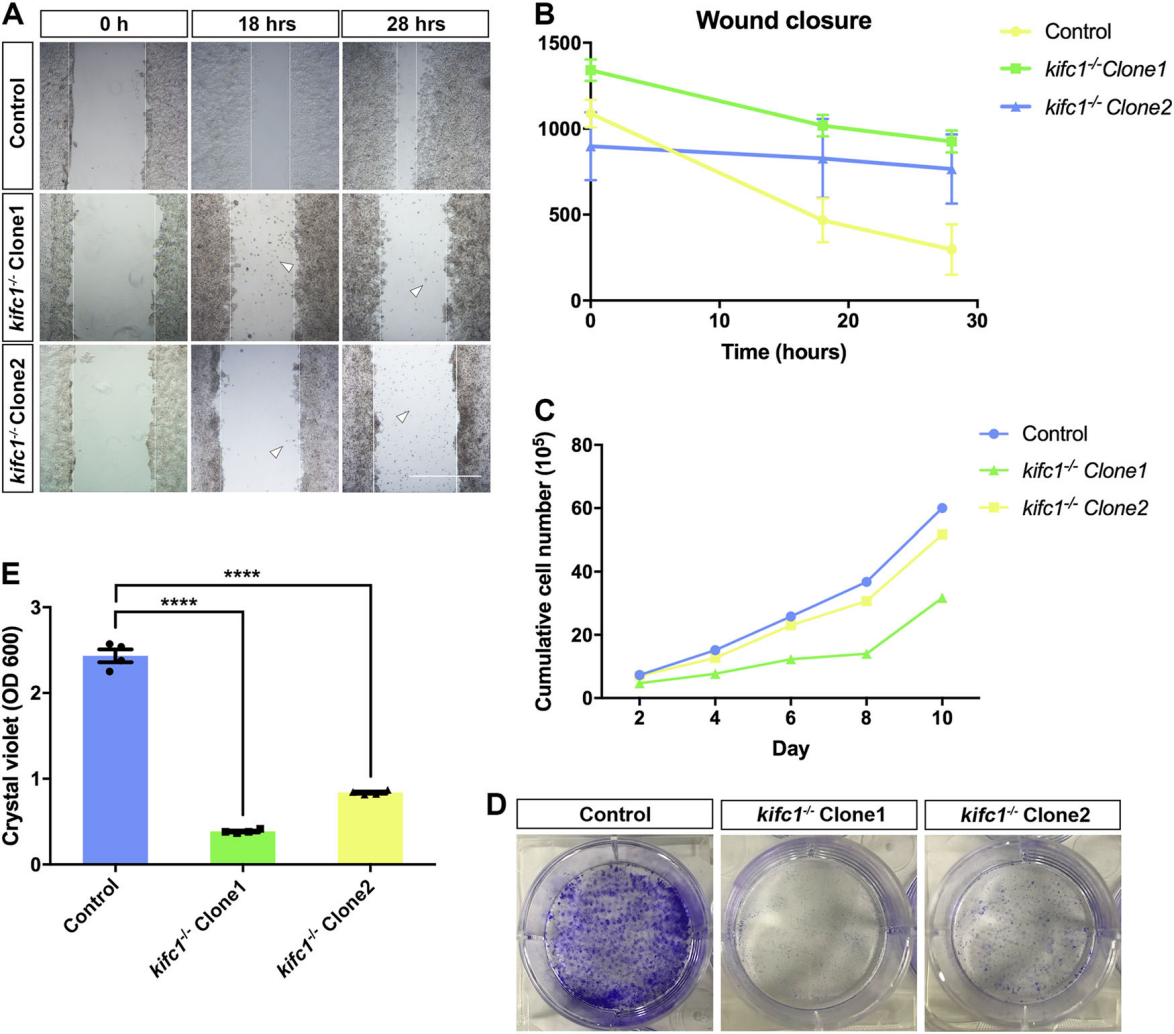
*kifc1* knockout inhibits the proliferation and growth of cells. **a** Cell wound healing assay of the two cell lines *kifc1*^*−/−*^ Clone1 and *kifc1*^*−/−*^ Clone2 healed from the scratched mechanical damage after 18 and 28 h. Large amounts of dead cells appeared between the closure (white arrowheads). Scale bar = 500 µm. **b** Statistical analysis of the rate of closure. **c** Growth curve analysis in *kifc1*^*−/−*^ cells and the control group. **d** Cell colony formation and **e** quantification, of the *kifc1* knockout cells. Two-tailed Student’s *t*-test; ****p < 0.0001; Error bars represent the mean ± S.E.M. *n* = 3

To further explore the potential functions of KIFC1 in cells, we used two cell lines which knockout different *kifc1* loci by CRISPR-Cas9 system in 293T cells (indicated as *kifc1*^*−/−*^ Clone1 and Clone2)^36^. In the process of cell culture, the cells grew slower in the absence of *kifc1* (Fig. 2c), and they form fewer cell colonies between cell aggregations. We suspected that it might relate to the involvements of KIFC1 in the transport of organelles or certain essential factors in cells. Therefore, we further explored the effects of *kifc1*^*−/−*^ cells in maintaining cell growth and proliferation. Through the wound healing assay, the normal control cells quickly heal from scratched mechanical damage and progress proliferation after 18 h (Fig. 2a). However, the cell damage was serious to heal over 28 h and large amounts of dead cells appeared between the closure with depletion of *kifc1* (Fig. 2a; white arrowheads). Similarly, when the cell colonies achieved a rich formation in normal cells, it is formidable for *kifc1*^−/−^ defects progress proliferation or cell confluence, and even most of the cell aggregations would quickly float off the plate and induce cell death (Fig. 2d). Consequently, we hypothesized that loss of KIFC1 motors might damage the intercellular connections or cell adhesive forces, thus results in the inhibition of cell proliferation.

### Depletion of KIFC1 causes S phase elongation

**Fig. 3:**
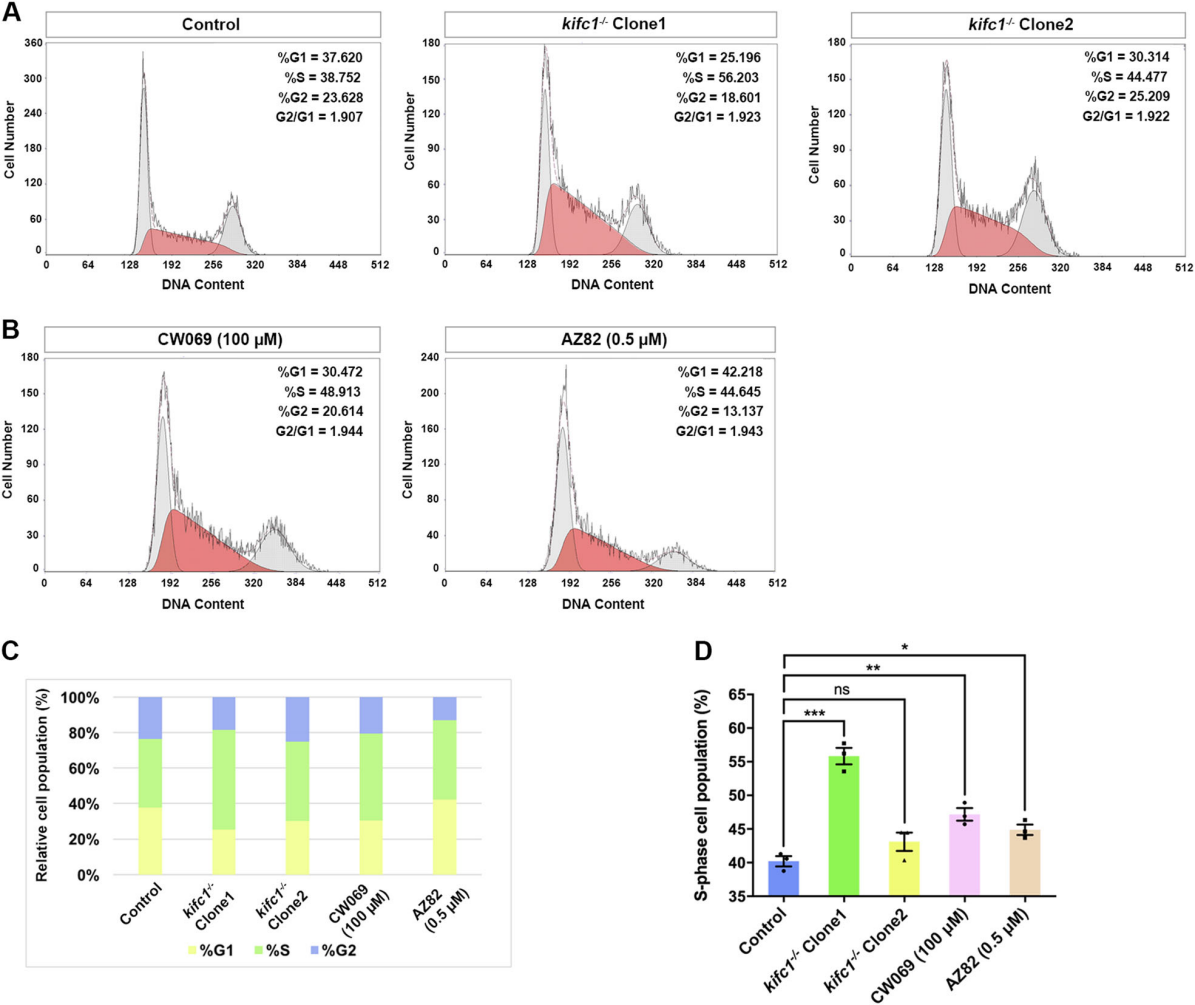
*kifc1*^*−/−*^ extends the S-phase of cell cycle. **a** Cell cycle analysis in two *kifc1* knockout cell lines and **b** in cells treated with (100 µM) CW069 and (0.5 µM) AZ82 inhibitors of KIFC1 through PI staining and flow cytometry. The significant differences of S-phase were identified by red region. **c, d** The relative population of cells in S-phase and the other phases of cell cycle under various conditions. Data are represented as mean ± S.E.M. with two-tailed Student’s *t*-test; *ns* not significant; *p < 0.05; **p < 0.01; ***p < 0.001; *n* = 3

To clarify the functions of KIFC1 in cell growth and DNA replication, we compared the alteration in the proportion of cell population without KIFC1 at different stages of cell cycle under the same conditions through propidium (PI) staining and flow cytometry (Fig. 3a). In the cell cycle of *kifc1*^*−/−*^ cell lines, though the number of cells distributed in G1 or G2 phase decreased obviously, the corresponding G1 and G2 phases had a certain fluctuation (Table S3), and a large number of cells resided in S phase (Fig. 3c, d). These cells also maintained normal DNA content, however, the relative cell population showed a 6–17% increase during DNA replication (Table S3). Here, we also selected two small molecular inhibitors for KIFC1: AZ82^35^ and CW069^34^. Cells treated with these inhibitors following a delayed growth rate but do not affect the cell division as mitotic abnormalities in *kifc1*-depleted cells. Similarly, the cells supplemented with each inhibitor according to the *K*_*i*_ of 0.5 and 100 µM separately also showed a 6–10% increase in relative cell number during S phase (Fig. 3b). Thus, KIFC1 is closely associated with the duration of interphase, especially S phase.

**Fig. 4:**
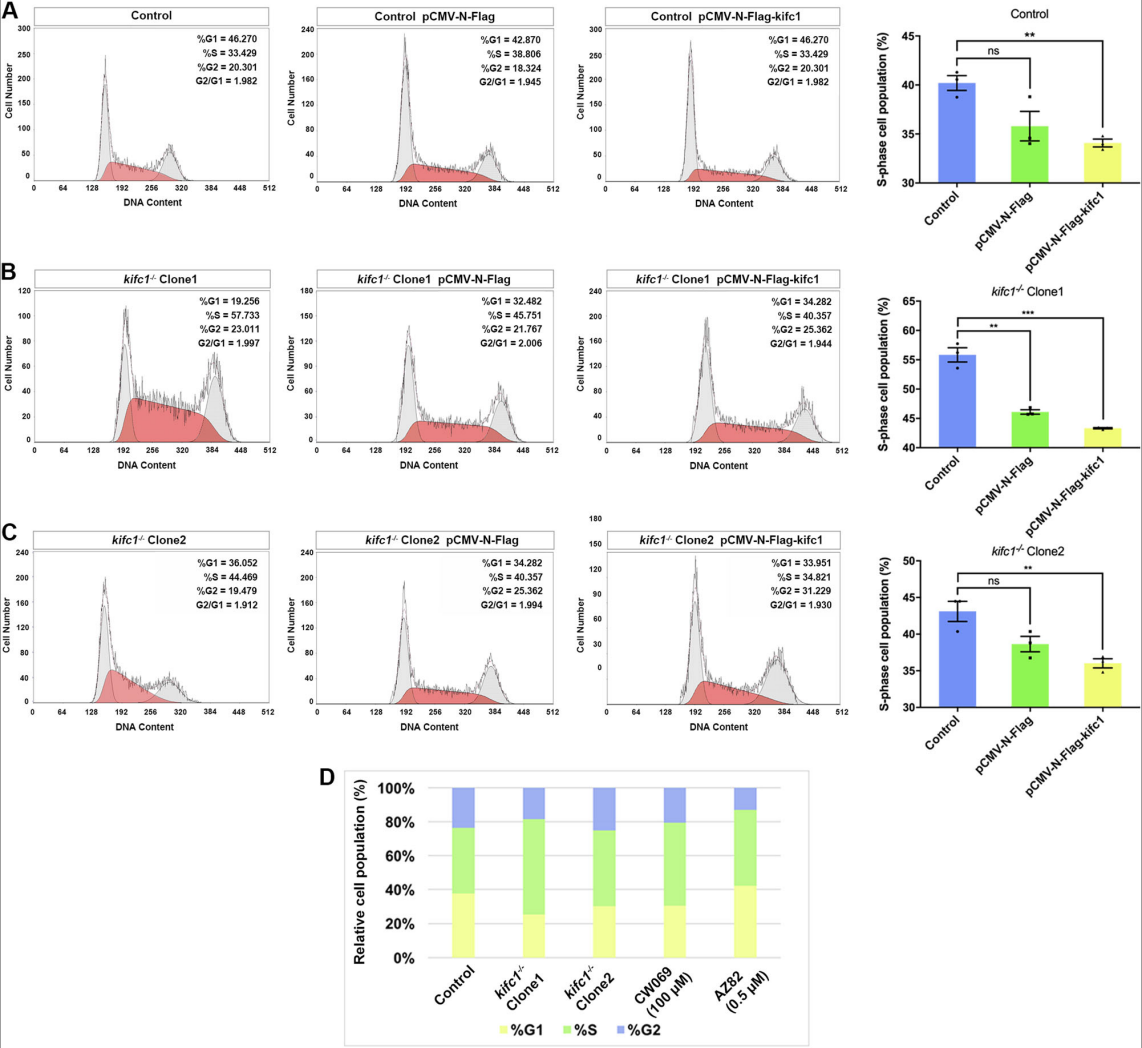
*kifc1* overexpression rescued the extended S-phase of cell cycle. **a–c** Full length of KIFC1 fusion protein was transfected in 293T cells and the *kifc1*^*−/−*^ cell lines for over 36 h then using PI staining and flow cytometry to analyze the corresponding cell cycle. The striking differences of S-phase were marked by red region and compared with the control ones. **d** The relative population of cells in each phases of cell cycle under various conditions. Data are represented as mean ± S.E.M. with two-tailed Student’s *t*-test; *ns* not significant; **p < 0.01; ***p < 0.001; *n* = 3

KIFC1 might regulate DNA replication through checkpoint activation and stalled the replication forks during S phase. This is one of the most predictable reasons why lack of KIFC1 can cause delayed cell proliferation. To identify whether KIFC1 is a key factor that postpone the DNA replication and defect the checkpoint activation, the full-length of human *kifc1* was cloned and constructed in a N-flag-tagged vector, then transfected to each cell line with effective expression compared to *β-actin* normalization (Fig. S1E). Intriguingly, after KIFC1 overexpression in 293T cells, the cells in S phase had a strikingly 5% decrease with an increased DNA content (Fig. 4a), while the elongated S phase of the two *kifc1*-knockout cell lines was also rescued to a certain extent and tended to be in a normal state. The relative cell numbers also had a 3–6% decrease (Fig. 4b–d). Taken together, these results demonstrated that KIFC1 proteins regulate DNA replication, and to some extent controls the occurrence of mitosis and maintaining genomic stability.

### KIFC1 plays a crucial role in nuclear membrane maintenance

**Fig. 5:**
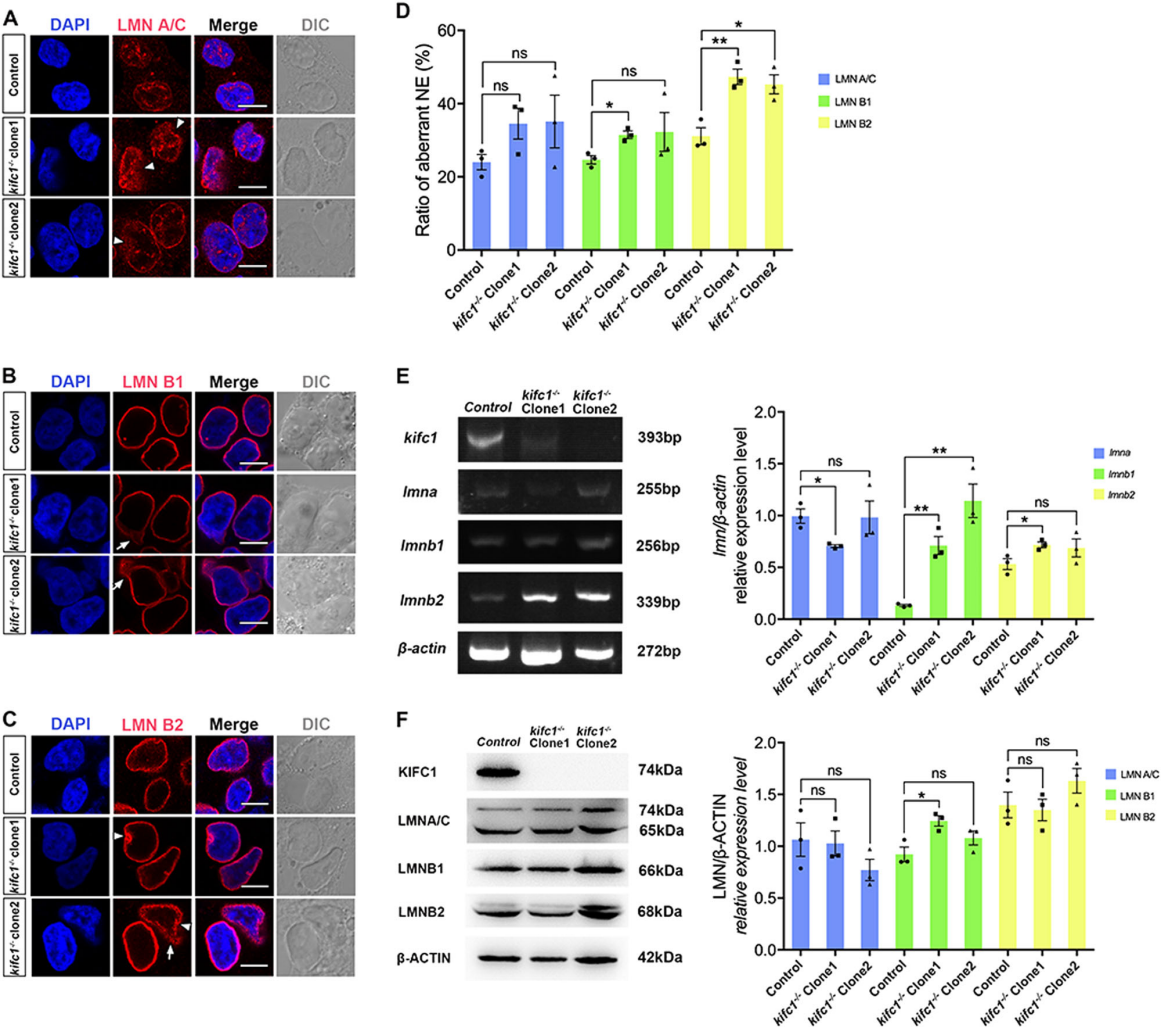
*kifc1*^*−/−*^ increases the ratio of aberrant nuclear lamina. **a–c** Confocol images of the position of major nuclear lamins LMN A/C, LMN B1 and LMN B2 in 293T cells and the *kifc1*^*−/−*^ cells. DAPI (blue), lamins (red). Scale bars = 5 µm. White arrowheads and white arrows represent the diffused and concaved nuclear lamina separately. **d** The quantified ratio of the aberrant nuclear envelope (NE) with defective lamins in different cell lines. Data represent the mean ± S.E.M. with two-tailed Student’s *t*-test; ns, not significant; *p < 0.05; **p < 0.01; *n* = 100. **e** Semi-quantitative RT-PCR and the quantification of each lamin genes in different cell lines. **f** Western blot and the quantification of each lamin proteins in different cell lines. *n* = 3

**Fig. 6:**
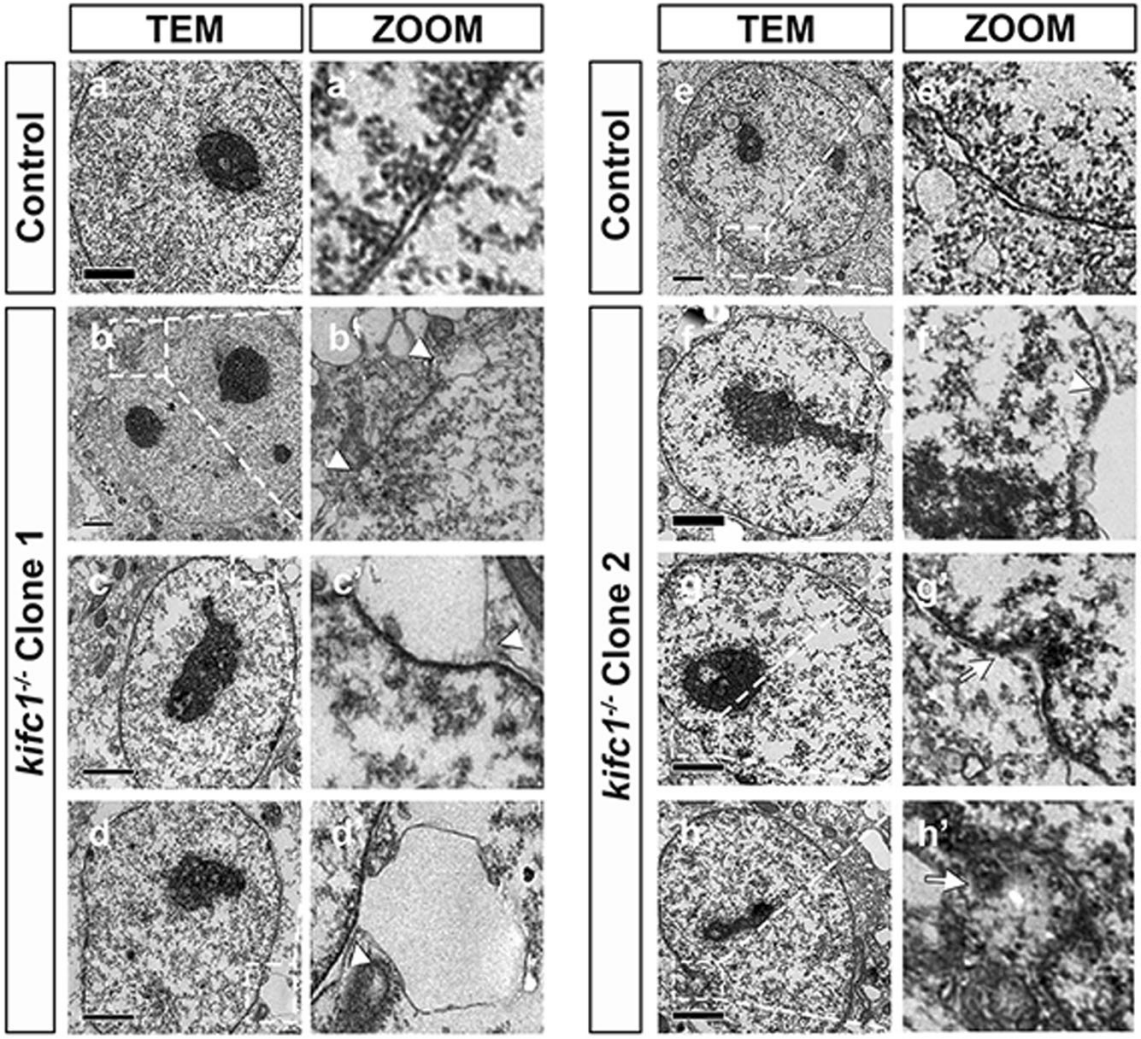
TEM assay shows the swell and breakage of nuclear envelope. **a**, **a’**; **e**, **e’** TEM images show the integrity of nuclear envelope and the corresponding magnification in 293T cells. **b-d**; **b’-d’** The damaged nuclear membrane in *kifc1*^*−/−*^ Clone1 cells, and **f-h**; **f’-h’** *kifc1*^−*/−*^ Clone2 cells. White arrowheads and white arrows represent the swelled and concaved nuclear membrane separately

We further detect if there were other cellular defects, particularly in nuclear morphology and the maintenance of nuclear membrane. Here, we detected a significant increase of B-type lamins in *kifc1*^−*/−*^ cells (Fig. 5e, f), and the immunofluorescence images showed that the nuclei with aberrant morphologies were simultaneous damaged the nuclear membrane with disordered lamins (Fig. 5a–c). The lamins diffused around the nucleus, either dispersed into the nucleoplasm, pulled the part of the nuclear lamina into the nucleus (Fig. 5c, white arrowheads), or flew to the cytoplasm, pushed the filamentous layer out with a protruding vesicle (Fig. 5b, c, white arrows). We counted hundreds of cells in each group and found that no matter which kind of defective lamins, they would show a higher ratio of aberrant nuclear membranes in *kifc1*^*−/−*^ cells (Fig. 5d). The magnified ultrastructure of the damaged nuclear membrane could appear explicitly using transmission electron microscopy assay (TEM). The lipid layer between the outer-inner nuclear membrane is obviously swelled, which protruded the outer nuclear membrane with a bubble-like enlargement (Fig. 6b’-f’, white arrowheads), while the inner nuclear membrane was subject to reverse extrusion with an obvious concave, even dispersed the fracture of nuclear membrane (Fig. 6g’, h’, white arrows). Thus, we considered that the nuclear phenotype of the *kifc1* knockout cells is severely damaged, and such damage is likely to cause a catastrophic imbalance of homeostasis to the internal and external nuclear membrane or block the orderly nucleo-cytoplasmic transportation. Besides, we found that there were many abnormal nucleoli in *kifc1*^*−/−*^ cells in interphase, which might also be related to the involvement of KIFC1 in DNA replication.

### KIFC1 regulates the distribution of chromatins in the nucleus

**Fig. 7:**
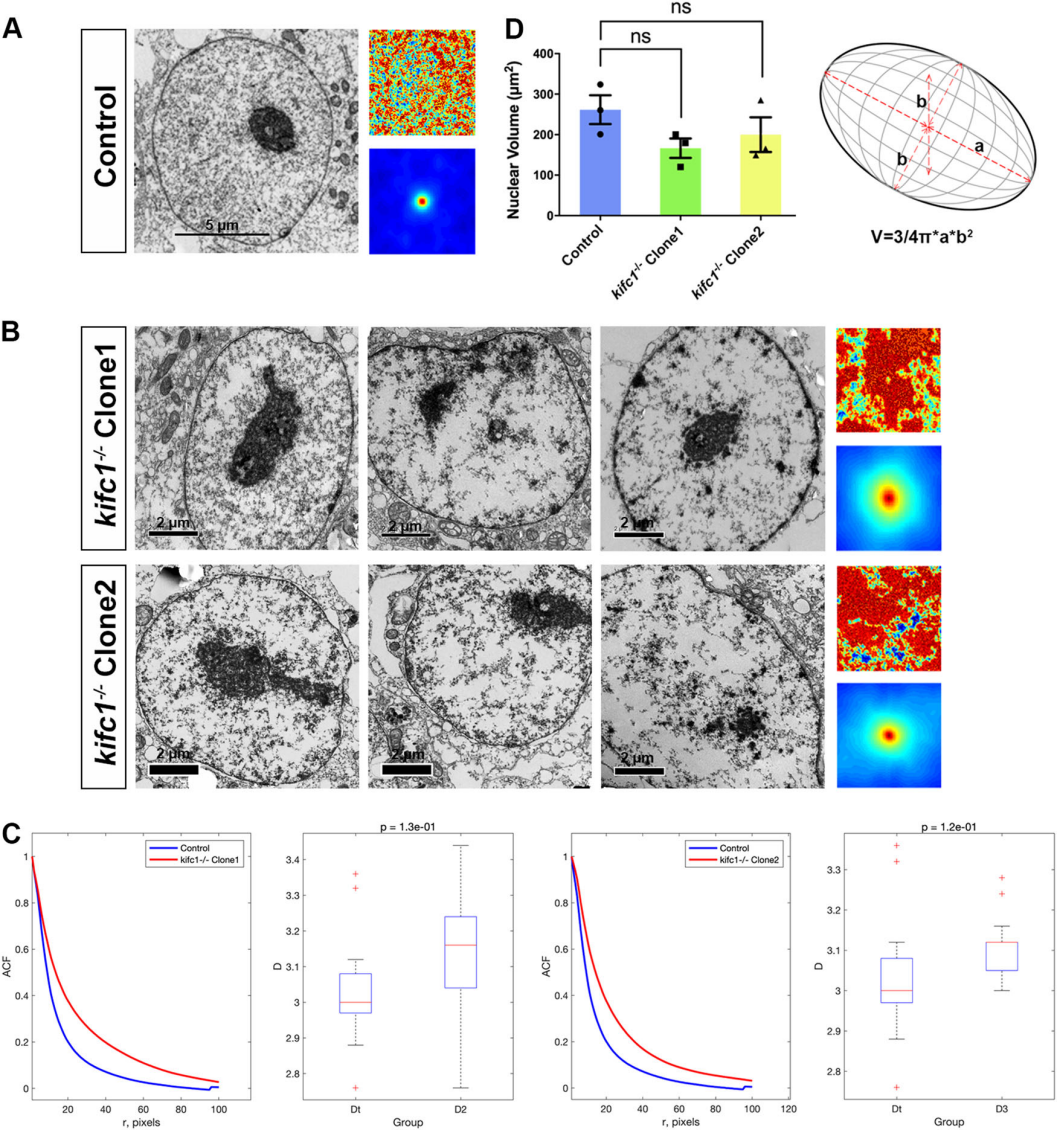
The abnormal morphologies of nucleolus and disordered chromatin density distribution in cells. **a-b** The measured separation *r* (pixel) and the original images (*n* = 5) for the degree of mass density correlation function in the chromatin of 293T cells and two *kifc1*^*−/−*^ cells. **c** Comparison of the spatial ACF and the *D*-value between *kifc1*^*−/−*^ cells and the control one. Boxplots indicate all values of *D* corresponding to the correlation functions. **d** Mathematical model for calculating the volume of the nucleus. Mean ± S.E.M. with two-tailed Student’s *t*-test; *ns* not significant; *n* = 3

The arrangement and package of the chromatin can directly affect the chromosomes segregation and gene expression, while the spatial distribution of chromatin in the nucleus can also regulate the size and the function of the nucleus. In this study, no significant differences were found in the size of the nuclei of each group (Fig. 7d). We could observe that in the normal nuclei, heterochromatins were uniformly distributed in the regions associated to the nucleoli and the inner nuclear membrane (i.e., nuclear lamina) (Fig. 7a). In contrast, the heterochromatins of the *kifc1*^*−/−*^ cells tended to wrap into several large clumps, and the chromatin in the nucleus was scattered (Fig. 7b). It has been considered as a reliable method to obtain the gray-value of images by digitizing the TEM micrograph and quantifying the chromatin density distribution through mathematical processes^37,38^, which reflects the proportion of the fragmented chromatin in the nucleus. To know the two-dimensional (2D) correlation function, we ran the existing codes from the script on github (https://github.com/barouxlab/ChromDensityNano) using the MATLAB software. Multiple images were successively obtained in each sample, after that the corresponding *D*-value and the 2D color-coded map were obtained based on the quantitative spatial autocorrelation function (ACF) of chromatin density of the nucleus (Fig. 7c). Specifically, the *D*-value of the control group, that is, the quantified shape of the mass density spatial correlation function tends to be between 2 and 3, implying that the chromatin distribution with a fracture nature. However, the *D*-value of the nucleus in the *kifc1*^*−/−*^ cells was increased significantly, showing that the chromatin had an increase in the width of the correlation function, with the larger and deeper chromatin clumps. Taken together, the results were likely to be associated with cell replication and the response to damage, thereby affecting the cell cycle.

### The ablation of kifc1 perturbs the chromosome architecture

**Fig. 8:**
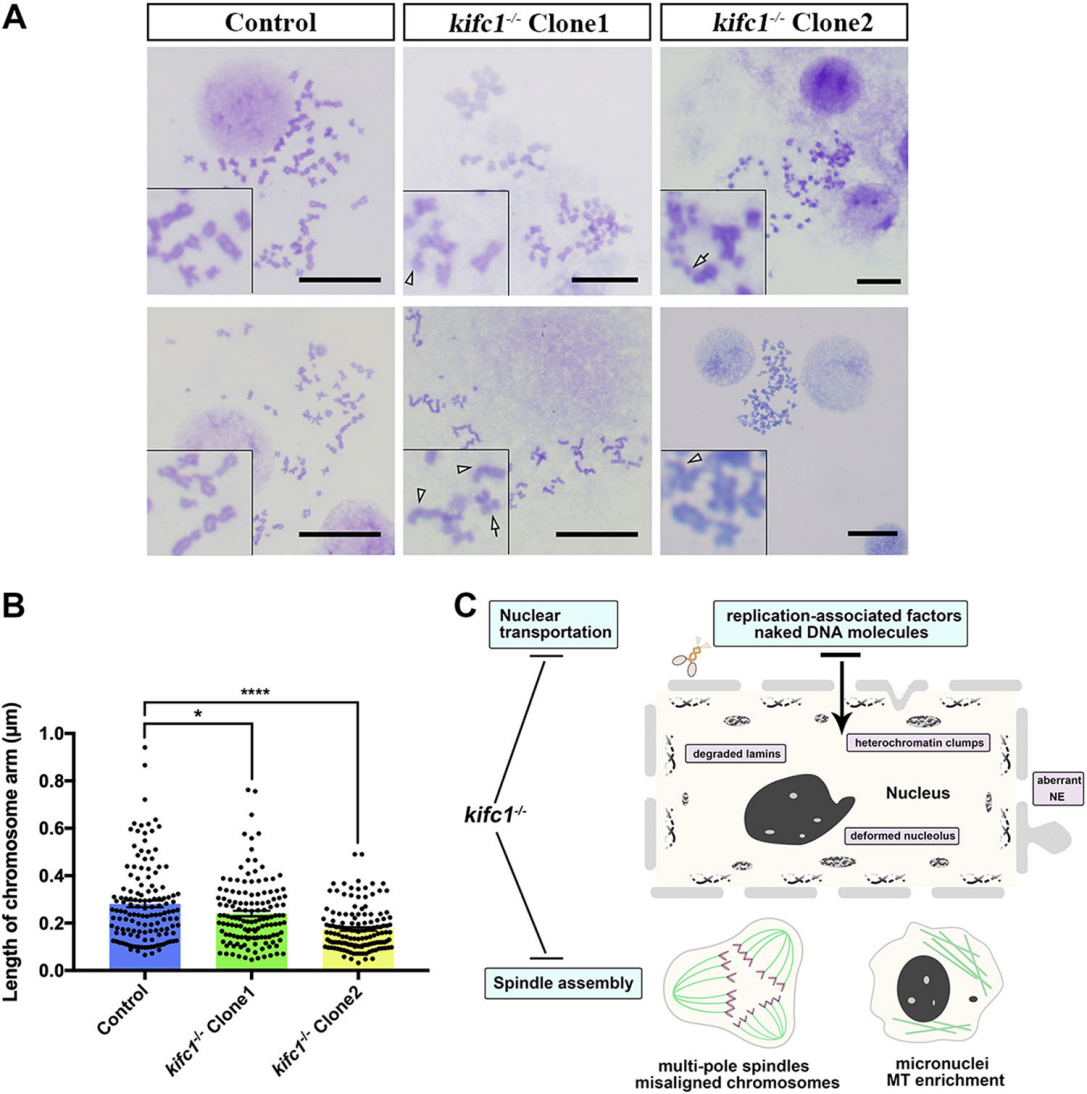
*kifc1* knockout misaligns the spindles during mitosis and the chromosomes. **a** Chromosome karyotype of 293T and *kifc1*^*−/−*^ cells with Giemmsa staining. White arrowheads and white arrows indicate the curve at the chromosome centromere and the chromosomal fragmentation separately. Scale bars = 5 µm. **b** Quantification of the length of chromosome arms. *n* = 100. Mean ± S.E.M. with two-tailed Student’s *t*-test; *p < 0.05; ****p < 0.0001. **c** Schemetic model of *kifc1*^*−/−*^ cells. Lack of KIFC1 proteins in somatic cells mainly disorders two crucial functions: the nuclear transportation and microtubule binding property. Consequently, the cytoplasmic replication-associated factors and DNA molecules cannot be transported into the nucleus, meanwhile, excessive microtubules would remain in cytoplasm and affect the cytoskeleton system, following the lamin degradation, heterochromatin clumps appearance, nucleolus and NE deformation. Thereafter, mitotic microtubules fail to assemble the normal spindles and arrange chromosomes in the correct way, even form the micronuclei after cytokinesis. *NE* nuclear envelope, *MT* microtubule

KIFC1 proteins are mainly involved in regulating the assembly and integration of spindle during mitosis. Consequently, it appeared many abnormal spindle structures in the *kifc1*^*−/−*^ cells, which altered the distance between the two poles of the cell and even produced multiple spindle poles (Fig. S1C), thus affecting the arrangement and separation of chromosomes. Such the aberrant cell division also accompanied by the appearance of micronucleus. To further explore the function in chromatin formation or chromosome segregation of KIFC1, we used colchicine to treat each group of cells, and the chromosomes in the metaphase were collected and analyzed the karyotypes (Fig. 8a). We found that the chromosome arms in the *kifc1* knockout cell lines were short (Fig. 8b) and mainly reflected a curve at the centromere (white arrowheads). Furthermore, it was accompanied by few of chromosome fragmentation and chromosomal gap (white arrows). Together, the disordered chromosome segregation was closely related to the involvement of KIFC1 in coordinating the spindle, however the delayed replication might increase the risk of aneuploidy formation.

## Discussion

Chromokinesins have been extensively studied for their nuclear localization properties and the proteins with NLS are mainly located within the nucleus during interphase. Besides the NLS, there is also a unique DNA binding region correlating with multiple chromatin integration and recombinant factors at S phase^39^. During mitosis, KIF4A proteins are closely bounded to the condensed chromosomes until cell division has completed, whereas the cells with depleted-KIF4A would not affect the intracellular microtubules and nuclear laminas^12^. On the contrary, we found that KIFC1 proteins were distributed almost throughout the cell during the interphase, and large number of the proteins appeared to transport into the nucleus when DNA replication occurred. The arrangement and assembly of microtubules can be disrupted after knock down of KIFC1^36,40,41^. Furthermore, the cells show a serious defect on proliferation with damaged nuclear membrane and laminas. The misaligned microtubules disturb the spindle integration thereafter accompany by forming several micronuclei (Fig. S1C). Plus, KIFC1 is different from most plus-end directed N-terminal kinesins. It binds, crosslinks and slides microtubules through the motor/tail-domain, which is crucial for regulating the aggregation of cellular cytoskeleton and coordinating the transport of essential factors to the minus-pole (Fig. S1D). The nuclear specific cyclin A only appears when DNA synthesis happens, and then disperses at the early stage of mitosis^42^. However, *kifc1* knockdown prolongs the prometaphase of cells and delays the degradation of cyclin A, then perturbs the chromosomal condensation and alignment, resulting in misaligned chromosomes, abnormal karyotype, and the formation of micronuclei^26,40,41,43^. Previously, Farina et al. found that in cell extracts, KIFC1 proteins could correlate with DNA motion and exclusively bound with the single nomadic DNA molecules ferrying along the microtubules through the motility assay in vitro^25^. In this study, we found that KIFC1 was involved in DNA replication or DNA damage repair mechanism as a potential factor. Specifically, cell cycle kinetics of *kifc1*-depleted cells were significantly reduced in the prolonged S phase and such damage was rescued after KIFC1 overexpression. In addition, we also found that KIFC1 overexpression in normal 293T cells not only shortened the duration of S phase, but also resulted in a certain amount of increased DNA content during replication. In other words, excess KIFC1 allows more DNA to be synthesized in a shorter S phase. Together, we revealed that KIFC1 proteins may have transported the cargos into nucleus for DNA replication and the postponement is conceivable related to DNA damage response, which can cause the block and fracture of the replication fork and activate the DNA repair and checkpoint pathways^44,45^. Another, the produced chromatin in the mid-/late-replication phase tend to locate at the periphery of the nuclear membrane^46,47^. Faulty or incomplete DNA replication will directly affect the condensation of chromatin and its spatial distribution within the nucleus.

Nuclear lamina provides mechanical support for the nuclear membrane, and all three lamins have chromatin anchoring sites. Among them, Lamin A/C also has the NLS in tail domain and is likely to participate in DNA replication, while Lamin B1 and Lamin B2 participate in the assembly of post-replication chromatin. During interphase, the chromatin is enriched around the nuclear lamina and nucleoli of cells and forms the heterochromatin regions^48–53^. If cell apoptosis occurs, the lamins gradually dissolve and degrade into the small fragments and form concentrated chromosomes^54^. For patients with Hutchinson-Gilford progeria syndrome (HGPS), one of the most significant defects is the mutation of the *lmna* gene. The non-functional lamins alter the trimethylated H3K27 binding site, affecting the amount and distribution of heterochromatin in the nucleus^55^. Here, we found that in the nucleus of the KIFC1 knockout cells, there were abnormal morphology of nucleoli, and the heterochromatin dispersed into condensed small clumps around the nuclear membrane as a response to DNA damage, especially with the aberrant density and increased interval of chromatin distribution in the nucleus^56^. All kinds of lamins show structural defects following the protein dispersion in the cytoplasm, and the outer/inner-nuclear membrane obviously forms the phenotype of expanded bulge or concaved depression. Taken together, we considered that after the ablation of *kifc1*, the cells could not maintain normal cell growth and proliferation, and would cause apoptosis. The KIFC1 overexpressed Hela cells resulted in an increased cell cycle kinetics, which is in consistent with our study. However, when knocked down the KIFC1, the delayed phase was G2/M rather than S phase in our study. It is known that the expression of KIFC1 in cancer cells is much more than that in wild types, which is mainly reflected in that KIFC1 motors interfere in spindle assembly in mitosis, forming multiple centrosomes, thus accelerating cell division^57^. However, the expression levels of KIFC1 in normal cells at different phases of the cell cycle is different.

In conclusion, we hypothesized that after the complete ablation of *kifc1*, DNA replication of cells is primarily affected, and the essential factors involved in the replication or free DNA molecules in the cytoplasm could not be normally transported into the nucleus by KIFC1, thus activating the DNA damage repair mechanism, leading to the prolongation of the S phase. After entering mitosis, *kifc1* deletion further affect the assembly of microtubules and mitotic spindles, increasing the occurrence of lagging chromosome and aneuploidy. Consequently, cell damage initiate apoptosis, and part of the lamins are degraded, which also affect the distribution of chromatin in the nucleus (Fig. 8b). In addition, KIFC1 proteins are overexpressed in many cancer samples and it is considered to be one of the crucial factors for driving the progression of tumors^58–60^. KIFC1 overexpression stimulates the cell colony formation and promotes cell migration^58^, which is consistent with our results that *kifc1* knockout severely affects cell proliferation. It was also found in a kinesin-like protein (Mklp-1) that the ectopic expression of NLS of Mklp-1 could cease cytokinesis^61^. It suggests that members of the kinesin superfamily may be potential proteins involved in regulating the cell cycle. To find the key proteins recruited by KIFC1, we screened some nuclei and mitotic related proteins that may be involved in KIFC1 interaction through mass spectrometry analysis. However, the expression level of these proteins did not show significant changes in the two *kifc1*^*-/-*^ cell lines, which might to some extent prove that *kifc1* was irreplaceable in the somatic cells.

## Materials and methods

### kifc1 knockout cell lines

Two *kifc1* knockout cell lines (*kifc1*^*−/–*^ Clone1 and *kifc1*^*−/−*^ Clone2) were generated in our previous study^36^. The sgRNAs were designed using an online CRISPR design tool (http://crispr.mit.edu/) according to the standard instruction^62^. Two Cas9-gRNAs ending with NGG (PAM sequence) targeting two different *kifc1* coding regions (Table S1). The guide sequences were annealed and then phosphorylated using T4 polynucleotide kinase (Takara, 2021S) and ligased into the pSpCas9 (BB) plasmid (Addgene, 42230) for sgRNA and Cas9 protein expression. We transiently transfected the pSpCas9 (BB) plasmids and then cultured for 36 h. The transfected cells were diluted and sub-cultured in 96-well plates to screen the monoclonal *kifc1* knockout cell. The monoclonal *kifc1* knockout cells were selected at 30 days and had cultured for many generations. A pair of sequencing primers were designed to validate the genomic deletion (Table S1). The normal 293T cells were used as the control group in the following experiments.

### Cell culture, transfection, and inhibitor treatment

The human 293T cells (ATCC, CRL-3216) and two *kifc1* knockout cell lines were cultured in a 37 °C incubator with 5% CO_2_ in standard cell culture medium (Dulbecco’s modified Eagle’s medium (DMEM) with 10% fetal bovine serum and 100 U/ml penicillin/streptomycin) (all from GIBCO, USA). The full length KIFC1 was cloned and inserted with *Bam*H I restriction site into a commercial expression vector (pCMV-N-Flag; Beyotime, D2722), and the plasmid DNA was transfected in cells at approximately 70% confluence using Lipo6000 reagent (Beyotime, C0526). According to the manufacturer’s instruction, after incubated the overexpressed/rescued cells for 36 h, we then harvested the cells for western blot and cell cycle analysis. For inhibitor treatment, the small molecule compounds CW069 (100 µM; Cayman, USA) and AZ82 (0.5 µM; Cayman, USA) were supplemented in cultural medium as previously described^34,35^.

### Antibodies

The commercial antibodies with specific use (IF, immunofluorescence; WB, western blot) in this study were listed as follows: KIFC1 rabbit monoclonal antibody (1:100, IF; 1:10000, WB; Abcam, ab172620), Lamin A + C rabbit monoclonal antibody (1:250, IF; 1:1000, WB; Abcam, ab108922), Lamin B1 rabbit monoclonal antibody (1:10000, WB; Abcam, ab133741), Lamin B2 rabbit monoclonal antibody (1:250, IF; 1:1000, WB; Abcam, ab151735), Lamin B1 mouse monoclonal antibody (1:50, IF; Santa Cruz, sc-365962), KIF4A rabbit monoclonal antibody (1:1000, WB; Abcam, ab124903), PRC1 rabbit monoclonal antibody (1:10000, WB; Abcam, ab51248), KIF20A rabbit polyclonal antibody (1:500, WB; Sangon, D222587), MAD1L1 rabbit polyclonal antibody (1:500, WB; Sangon, D120938), MAD2L1 rabbit polyclonal antibody (1:200, WB; Sangon, D220939), PROX1 rabbit polyclonal antibody (1:500, WB; Sangon, D162082), hnRNP M1-4 mouse monoclonal antibody (1:200, WB; Santa Cruz, sc-20002), nucleoporin p62 mouse monoclonal antibody (1:200, WB; Santa Cruz, sc-48373), Histone H2A.X mouse monoclonal antibody (1:200, WB; Santa Cruz, sc-517336), Histone H2B mouse monoclonal antibody (1:200, WB; Santa Cruz, sc-515808), ACTB rabbit polyclonal antibody (1:1000, WB; BBI, D110001), secondary Alexa Fluor 555-conjugated donkey-anti-rabbit antibody (1:500, IF; Beyotime, A0453), secondary Alexa Fluor 555-conjugated donkey-anti-mouse antibody (1:500, IF; Beyotime, A0460), anti-Flag mouse monoclonal antibody (1:1000, WB; Beyotime, AF519-1), secondary goat-anti-rabbit HRP-conjugated antibody (1:2000, WB; Beyotime, A02028), secondary goat-anti-mouse HRP-conjugated antibody (1:2000, WB; BBI, D110087).

### Immunofluorescence

12 mm diameter coverglass (Fisherbrand) was sterilized and pretreated with 1% gelatin (Sangon, A609764) in a 24-well culture plate for 30 min, and then prepared for seeding the cells. After the cells grew to 80% confluence, it was fixed in 4% paraformaldehyde in phosphate buffered saline (PBS) for 15 min and rinsed with PBS for three times. Then the cells were permeabilized with 0.25% Triton X-100 in PBS for 10 min and followed by blocking in PBST solution (1% BSA and 0.05% Tween-20 in PBS) for one h at room temperature. The primary antibodies were diluted in PBST solution and incubated overnight at 4 °C. Respectively, the appropriate secondary antibodies were diluted (in PBST) and applied for one h at room temperature. Each step was completed with 4 times PBS washing. DAPI (Beyotime, C1005) was used to present the nucleus and treated for 5 min. The cells were final mounted with anti-fade mounting medium (Beyotime, P0126) and detected immediately using a confocal laser scanning microscope (Carl Zeiss, CLSM 710). The negative control samples were treated without primary antibodies.

### Semi-quantitative RT-PCR

Total RNAs of the cells were extracted with RNAiso Plus reagent (TaKaRa, 9108), and transcribed into cDNA reversely using the PrimeScript^TM^ RT Master Mix kit (TaKaRa, RR036). Pairs of gene specific primers (Table.S1) were designed and synthesized on Primer-Blast (https://www.ncbi.nlm.nih.gov/tools/primer-blast). The PCR procedure was set as follows: 98 °C for 10 s, 32 cycles of 98 °C for 10 s, 55 °C for 30 s, 72 °C for 30 s; 72 °C for 8 min. DNA band densities were detected with a 2% agarose gel, and then measured, normalized with relative *β-actin* mRNA expression level.

### Western blot

Cells werehomogenized and lysed in RIPA buffer (Beyotime, P0013B) containing 1% protease inhibitors (CWBIO, CW2200S). After boiled and denatured the sample in SDS-buffer, the equal amount of proteins was separated in 10% SDS-polyacrylamide gels by electrophoresis, and then transferred to polyvinylidene difluoride (PVDF) membranes (Millipore). The 5% non-fat milk in 0.1% TBST buffer was used to block the membrane for 1 h. The individual primary antibodies were diluted with blocking buffer and incubated for 12 h at 4 °C. The proper secondary HRP-conjugated antibodies were applied for one h. In these processes, the membrane was washed thoroughly in TBST buffer for three times. The blots of target protein were detected by an enhanced chemiluminescent kit (Beyotime, P0018FFT) and quantified the fold change with respect to *β-actin* normalization.

### Cell wound healing

A cell wound healing assay was used to detect the cell proliferation of different cell lines. After the cells growing to full confluency in the 6-well plates, the culture medium was changed to a non-serum medium and kept culture for 2 h. A 100 µl (middle size) micropipette was used to scratch the cells in a straight way. Afterwards, the medium was replaced to the standard system and kept culturing for 18 and 28 h. The wound area at each time point was captured and measured the rate of closure using ImageJ software (ImageJ, NIH). A linear fit was generated with GraphPad Prism 7.0 software as the comparison of cell proliferation. To determine the correlation of linear regression, the R^2^ was obtained from the mean of all Y values.

### Growth curve and colony-formation assay

For growth curve measurement, 200,000 cells of each cell line were seeded on 6-well plate and kept culturing in the incubator for 10 days. The cell number was measured every two days manually with the counting chamber (INCYTO, Korea), following the standard procedures.

For colony-formation, 2000 cells of each cell line were seeded on 6-well plate and kept culturing in the incubator for three weeks. The cell colonies were fixed by 1% paraformaldehyde in PBS and stained with 0.1% crystal violet (all for 15 min). The violet dye was eluted with 10% acetic acid and quantified OD600 using a Nanodrop-2000 spectrophotometer (Thermo Scientific).

### Cell cycle and EdU analysis

After removed the cells with 0.25% trypsin (GIBCO), the cell suspension was rinsed by pre-cooled PBS and then gently re-suspended with 70% ethanol at 4 °C for 12 h. The fixed cells were rinsed with PBS and incubated with propidium iodide containing RNase A at 37 °C for 30 min (Beyotime, C1052). The single cells were screened before flow analysis (Cytomic FC 500 MCL, Beckman Coulter, USA). Data was obtained using CXP software v2.2 (Beckman Coulter, USA), and set the parameter to FL3-620nm BP. 10,000 events were collected per sample manually determined the percentage of G1, G2 and S phases. Data in different groups were imported in Excel software (Microsoft) and generated the graph of relative cell population.

The S phase cells were detected by EdU Cell Proliferation Kit with Alexa Fluor 488 (Beyotime, C0071S). The cells on coverslip were treated with pre-warmed EdU (20 µM) for 2 h, and then fixed by 4 % paraformaldehyde in PBS for 15 min and permeabilized with 0.25% Triton X-100 in PBS for 10 min. Each process followed by PBS washing for 3 times and the cells were incubated in click additive solution (Click Reaction Buffer, CuSO_4_ and Azide 488) at room temperature for 30 min. The followed steps of staining the antibodies and nucleus were same as the immunofluorescence assay.

### Karyotype analysis

After treated the cells with colchicine (final concentration 0.3 μg/mL; Urchem, 61001582) for 8 h, the cells were trypsinized and resuspended in 0.075 M KCl solution at 37 °C for 30 min. Afterwards, the cells were fixed (in methanol/acetic acid 3:1) for two times and mounted onto pre-chilled slides, then stained with Giemsa for 10 min. The chromosomes were observed using a light microscope (Olympus BX 40).

### Transmission electron microscopic (TEM) assay

To determine the complementary ultrastructure of the nucleus and the chromatin distribution, a TEM analysis was performed with glutaraldehyde-osmic acid double fixation. The cells were trypsinized and centrifuged to a pellet, then fixed in 2.5% glutaraldehyde in PBS at 4 °C overnight. After PBS washing, the cells were post fixed in 1% osmic acid at room temperature for 1 and stained for 30 min with 2% uranyl acetate. After a sequential ethanol dehydration, the specimen was embedded in epoxy resin and cut to 50 nm thin sections. The lead citrate stained cells were photographed by TEM (Philips-FEI Tecnai T10, USA) and operated at 100 kV. The distribution of chromatin was quantified using the scripts from github (https://github.com/barouxlab/ChromDensityNano) on Matlab software and analyzed the qualitative changes in density autocorrelation functions (ACFs) of collected images.

### Statistical analysis

All experiments in this study were performed in triplicate and generated the data into GraphPad Prism 7.0 software to export the images with means ± SEM of different groups. Specifically, ns represents not significant, and p-values ≤ 0.05 were considered significant (*p-value < 0.05; **p-value < 0.01; ***p-value < 0.001).

## Supplementary information

Table S1

Table S2

Table S3

Figure S1

Supplementary figure legends
