# Supplementary material for: Kinesin-14 motor protein KIFC1 participates in DNA synthesis and chromatin maintenance
Source: Cell Death Dis. 2019 May 24;10(6):402. doi: 10.1038/s41419-019-1619-9 (PMC6534603; doi:10.1038/s41419-019-1619-9)
Supplement: Supplementary file 1 — Table S1 [file 41419_2019_1619_MOESM1_ESM.docx]

Table S1. List of specific primers

| Gene | Forward primer | Reverse primer | Function |
| --- | --- | --- | --- |
| *kifc1* | CAGACAGGCAGTGGCAAGACC | TACACTGTGGCTGCGTGATGACC | Semi-quantitative RT-PCR |
| *lmna* | AATGATCGCTTGGCGGTCTAC | CTTCTTGGTATTGCGCGCTTT |  |
| *lmnb1* | AAAAGACAACTCTCGTCGCAT | CCGCTTTCCTCTAGTTGTACG |  |
| *lmnb2* | ATTCAGAATCCAGGCGTCGAC | TTATTGTTGTGACAGGTCTTACGACG |  |
| *β-actin* | GATTCCTATGTGGGCGACGA | GATAGCACAGCCTGGATAGCA |  |
| *sequencing* | GTGAGAGGCTGGGATAGGGA | CCCTCCGTTCTTCCTGCAAT | *kifc1* knockout validation |
| *sgkifc1-1* | 5′-AACTAAAACGGTGCCGTGAG-3′ | | guide sequence |
| *sgkifc1-2* | 5′-CCGTGAGAGGACTCAAACGT-3′ | |  |
