# Supplementary material for: Kinesin-14 motor protein KIFC1 participates in DNA synthesis and chromatin maintenance
Source: Cell Death Dis. 2019 May 24;10(6):402. doi: 10.1038/s41419-019-1619-9 (PMC6534603; doi:10.1038/s41419-019-1619-9)
Supplement: Supplementary file 2 — Table S2 [file 41419_2019_1619_MOESM2_ESM.docx]

Table S2. Statistical analysis of the rate of closure.

|  | Control | *kifc1^-/-^* Clone1 | *kifc1^-/-^* Clone2 |
| --- | --- | --- | --- |
| Rate of closure (µm/h) | 28.286 | 14.809 | 4.696 |
| R-squared | 0.9764 | 0.9785 | 0.9866 |

* R-squared were obtained from the mean of each Y values.
