# Supplementary material for: Kinesin-14 motor protein KIFC1 participates in DNA synthesis and chromatin maintenance
Source: Cell Death Dis. 2019 May 24;10(6):402. doi: 10.1038/s41419-019-1619-9 (PMC6534603; doi:10.1038/s41419-019-1619-9)
Supplement: Supplementary file 3 — Table S3 [file 41419_2019_1619_MOESM3_ESM.docx]

Table S3. The relative population of cells in S-phase and the other phases of cell cycle under various conditions.

|  | %G1 | %S | %G2 | %G2/G1 |
| --- | --- | --- | --- | --- |
| Control | 37.62 | 38.752 | 23.628 | 1.907 |
| *kifc1^-/-^* Clone1 | 25.196 | 56.203 | 18.601 | 1.923 |
| *kifc1^-/-^* Clone2 | 30.314 | 44.477 | 25.209 | 1.922 |
| CW069 (100 µM) | 30.472 | 48.913 | 20.614 | 1.944 |
| AZ82 (0.5 µM) | 42.218 | 44.645 | 13.137 | 1.943 |

| Cell line | plasmid | %G1 | %S | %G2 | %G2/G1 |
| --- | --- | --- | --- | --- | --- |
| Control | pCMV-N-Flag | 42.87 | 38.806 | 18.324 | 1.945 |
| Control | pCMV-N-Flag-kifc1 | 46.27 | 33.429 | 20.301 | 1.982 |
| *kifc1^-/-^* Clone1 | pCMV-N-Flag | 32.482 | 45.751 | 21.767 | 2.006 |
| *kifc1^-/-^* Clone1 | pCMV-N-Flag-kifc1 | 36.623 | 43.108 | 20.269 | 2.005 |
| *kifc1^-/-^* Clone2 | pCMV-N-Flag | 34.282 | 40.357 | 25.362 | 1.994 |
| *kifc1^-/-^* Clone2 | pCMV-N-Flag-kifc1 | 33.951 | 34.821 | 31.229 | 1.93 |
