# Supplementary figures and images for: Kinesin-14 motor protein KIFC1 participates in DNA synthesis and chromatin maintenance
Source: Cell Death Dis. 2019 May 24;10(6):402. doi: 10.1038/s41419-019-1619-9 (PMC6534603; doi:10.1038/s41419-019-1619-9)

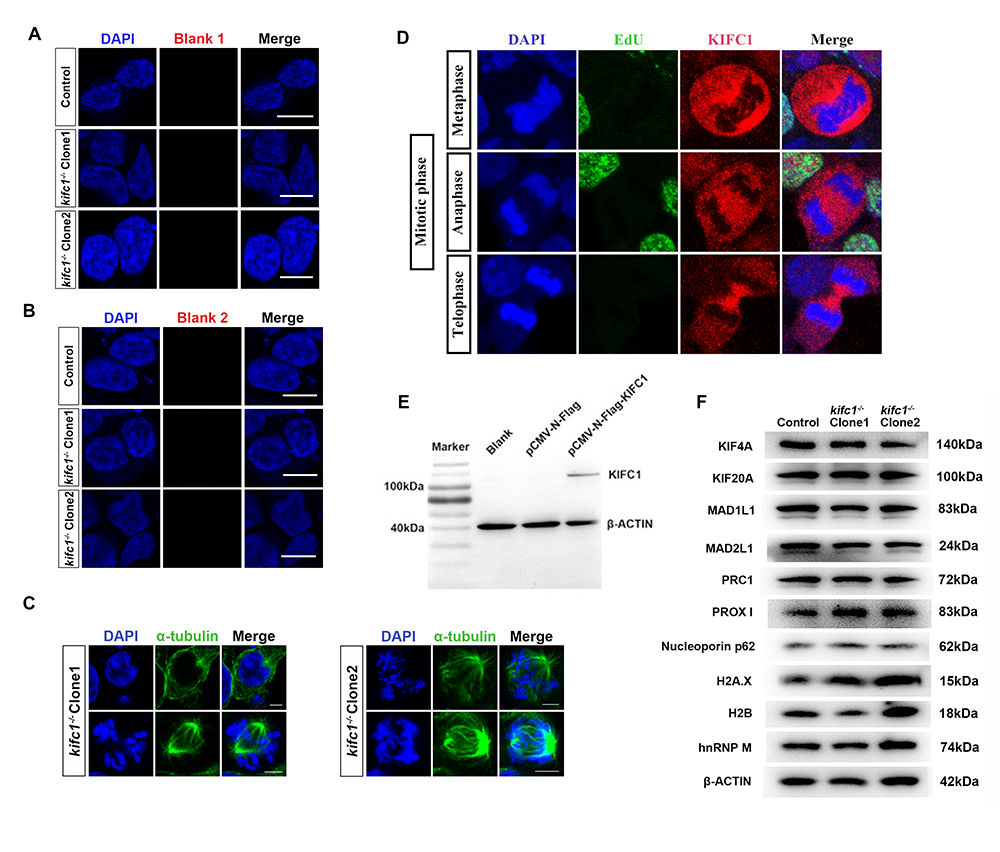

Supplement: Supplementary file 4 — Figure S1 [file 41419_2019_1619_MOESM4_ESM.tif]
