# Supplementary material for: Kinesin-14 motor protein KIFC1 participates in DNA synthesis and chromatin maintenance
Source: Cell Death Dis. 2019 May 24;10(6):402. doi: 10.1038/s41419-019-1619-9 (PMC6534603; doi:10.1038/s41419-019-1619-9)
Supplement: Supplementary file 5 — Supplementary figure legends [file 41419_2019_1619_MOESM5_ESM.docx]

**Supplementary Material Legends**

**Table S1. List of specific primers.**

**Table S2. Statistical analysis of the rate of closure.**

**Table S3. The relative population of cells in S-phase and the other phases of cell cycle under various conditions.**

**Fig S1. Negative controls and the KIFC1 potential relative proteins.**

(A) Negative control of secondary Alexa Fluor 555-conjugated donkey-anti-rabbit antibody and (B) 555-conjugated donkey-anti-mouse antibody for immunofluorescence in different cell lines. DAPI (blue), negative control (red). Scale bars = 5 µm. (C) Confocol images of *kifc1^-/-^* cells with misaligned, multipole spindles and the micronuclei. DAPI (blue), α-tubulin (green). Scale bars = 5 µm. (D) The spatio-temporal localization of KIFC1 (red) during mitotic phase. (E) Transfect efficiency of the full length KIFC1 fusion protein in 293T cells. (F) Western blot of KIFC1 potential relative proteins from the database of mass spectrometry.
